# Supplementary material for: Integrative Metabolic and Transcriptomic Profiling in Camellia oleifera and Camellia meiocarpa Uncover Potential Mechanisms That Govern Triacylglycerol Degradation during Seed Desiccation
Source: Plants (Basel). 2023 Jul 8;12(14):2591. doi: 10.3390/plants12142591 (PMC10385360; doi:10.3390/plants12142591)
Supplement: Supplementary file 1 [file plants-12-02591-s001.zip › Figure S2.pptx]

## Slide 1
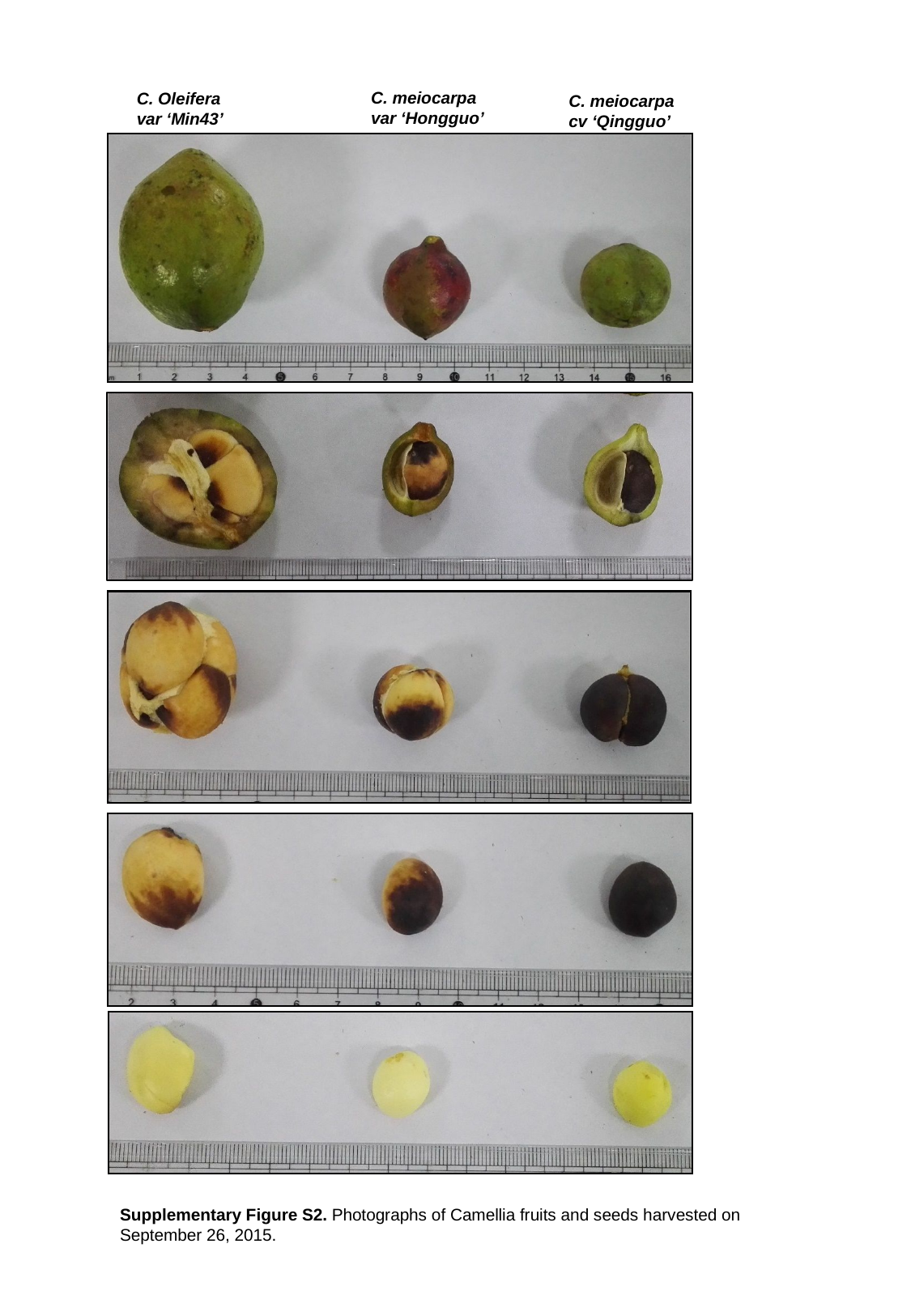

C. meiocarpa
var ‘Hongguo’
C. Oleifera
var ‘Min43’
C. meiocarpa
cv ‘Qingguo’
Supplementary Figure S2. Photographs of Camellia fruits and seeds harvested on September 26, 2015.
