# Supplementary material for: Integrative Metabolic and Transcriptomic Profiling in Camellia oleifera and Camellia meiocarpa Uncover Potential Mechanisms That Govern Triacylglycerol Degradation during Seed Desiccation
Source: Plants (Basel). 2023 Jul 8;12(14):2591. doi: 10.3390/plants12142591 (PMC10385360; doi:10.3390/plants12142591)
Supplement: Supplementary file 1 [file plants-12-02591-s001.zip › Figure S3.pptx]

## Slide 1
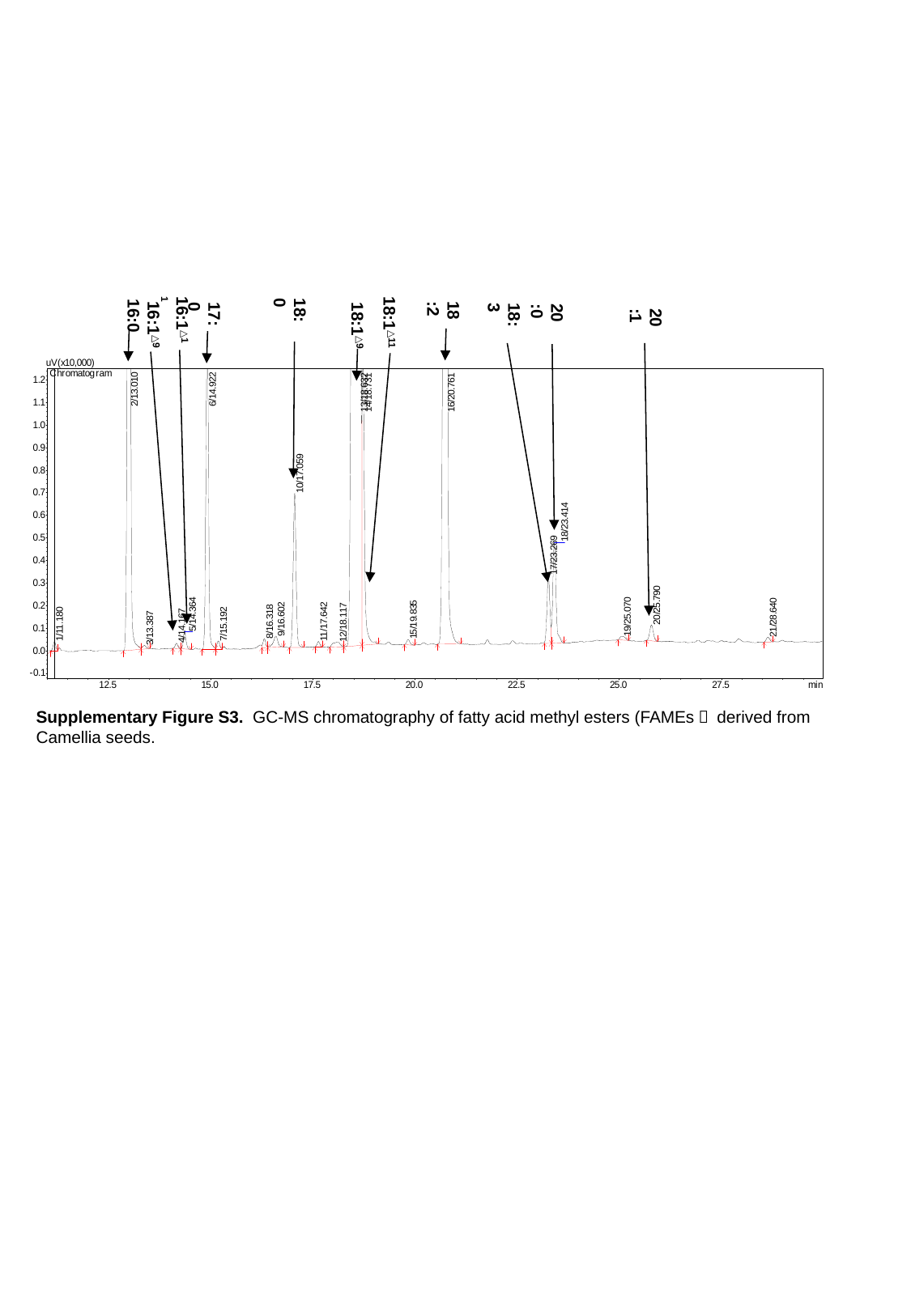

16:1△11
18:1△11
18:0
16:0
18:2
16:1△9
17:0
18:1△9
18:3
20:0
20:1
Supplementary Figure S3. GC-MS chromatography of fatty acid methyl esters (FAMEs） derived from Camellia seeds.
