# Supplementary material for: Integrative Metabolic and Transcriptomic Profiling in Camellia oleifera and Camellia meiocarpa Uncover Potential Mechanisms That Govern Triacylglycerol Degradation during Seed Desiccation
Source: Plants (Basel). 2023 Jul 8;12(14):2591. doi: 10.3390/plants12142591 (PMC10385360; doi:10.3390/plants12142591)
Supplement: Supplementary file 1 [file plants-12-02591-s001.zip › Figure S4.pptx]

## Slide 1
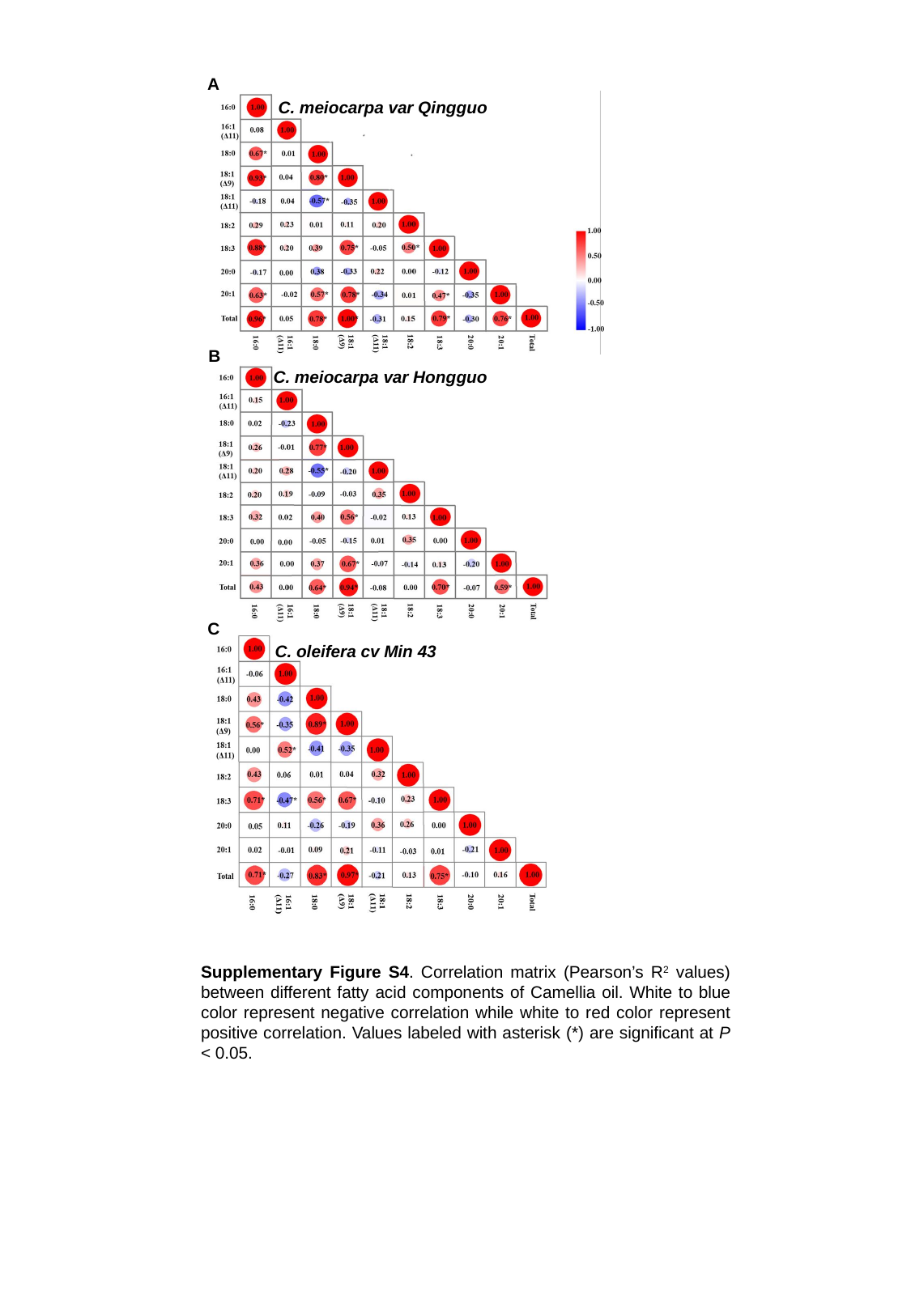

A
C. meiocarpa var Qingguo
B
C. meiocarpa var Hongguo
C
C. oleifera cv Min 43
Supplementary Figure S4. Correlation matrix (Pearson’s R2 values) between different fatty acid components of Camellia oil. White to blue color represent negative correlation while white to red color represent positive correlation. Values labeled with asterisk (*) are significant at P < 0.05.
