# Supplementary material for: Integrative Metabolic and Transcriptomic Profiling in Camellia oleifera and Camellia meiocarpa Uncover Potential Mechanisms That Govern Triacylglycerol Degradation during Seed Desiccation
Source: Plants (Basel). 2023 Jul 8;12(14):2591. doi: 10.3390/plants12142591 (PMC10385360; doi:10.3390/plants12142591)
Supplement: Supplementary file 1 [file plants-12-02591-s001.zip › Figure S6.pptx]

## Slide 1
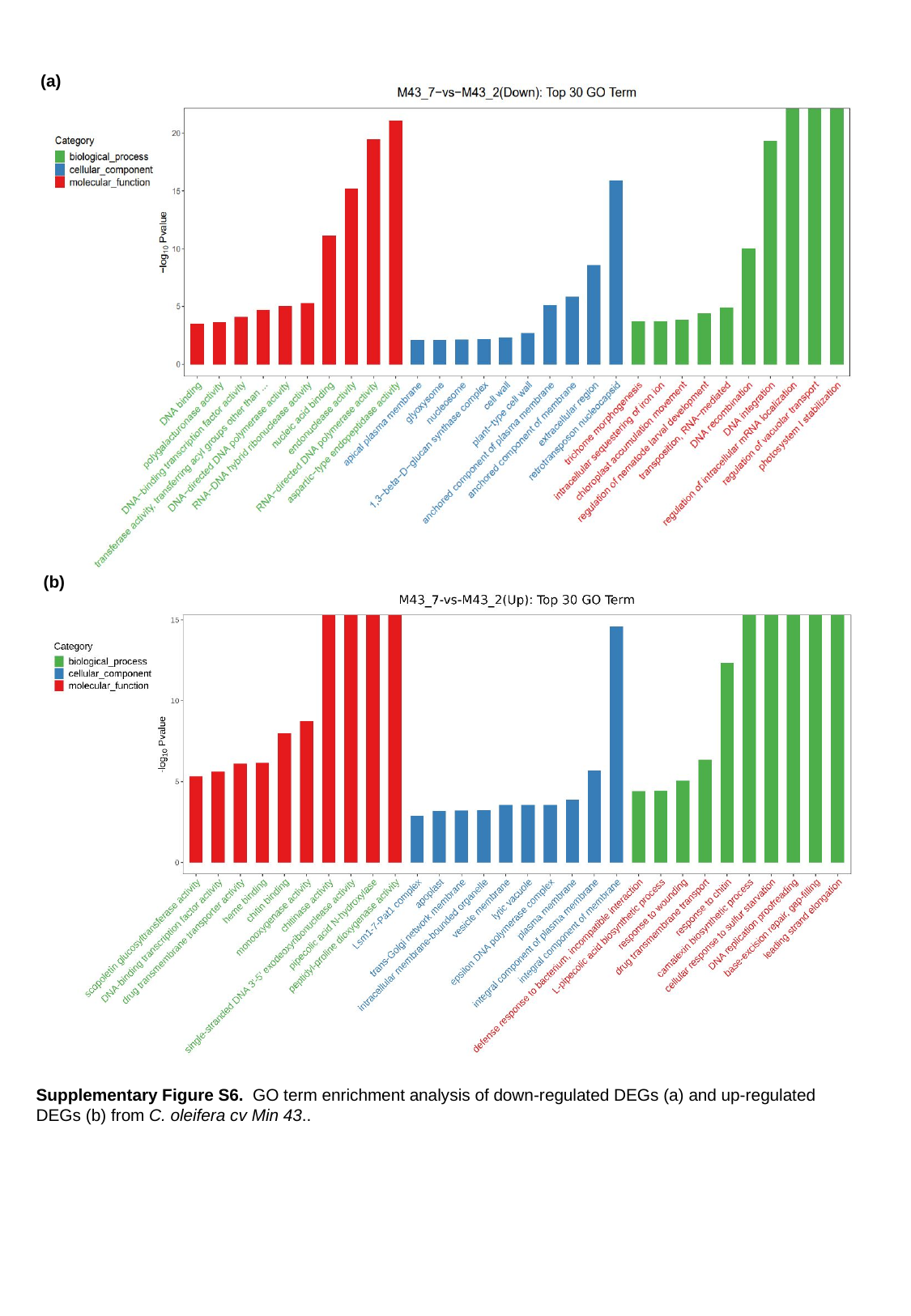

(a)
(b)
Supplementary Figure S6. GO term enrichment analysis of down-regulated DEGs (a) and up-regulated DEGs (b) from C. oleifera cv Min 43..
