# Supplementary material for: Integrative Metabolic and Transcriptomic Profiling in Camellia oleifera and Camellia meiocarpa Uncover Potential Mechanisms That Govern Triacylglycerol Degradation during Seed Desiccation
Source: Plants (Basel). 2023 Jul 8;12(14):2591. doi: 10.3390/plants12142591 (PMC10385360; doi:10.3390/plants12142591)
Supplement: Supplementary file 1 [file plants-12-02591-s001.zip › Figure S7.pptx]

## Slide 1
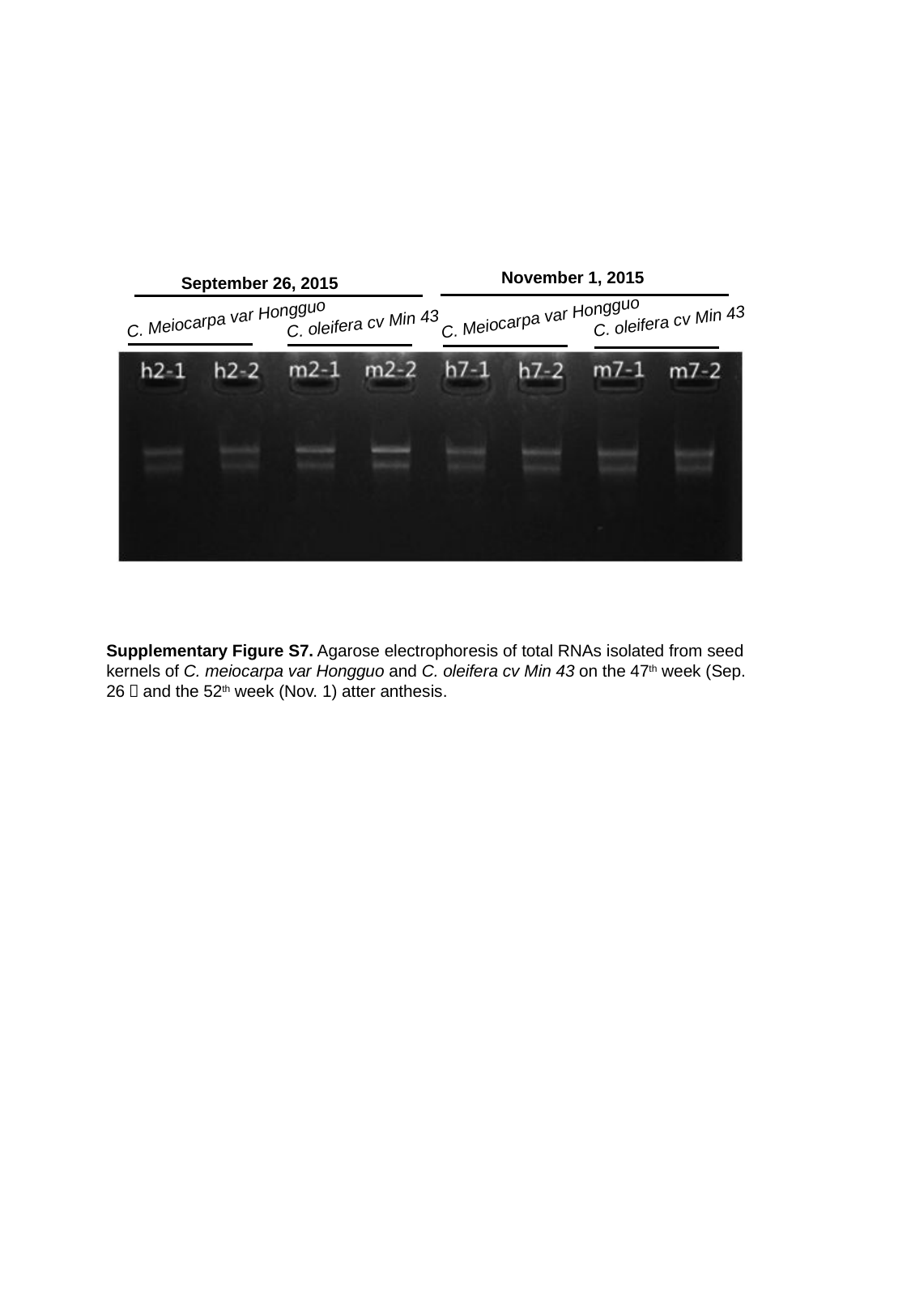

November 1, 2015
September 26, 2015
C. Meiocarpa var Hongguo
C. Meiocarpa var Hongguo
C. oleifera cv Min 43
C. oleifera cv Min 43
Supplementary Figure S7. Agarose electrophoresis of total RNAs isolated from seed kernels of C. meiocarpa var Hongguo and C. oleifera cv Min 43 on the 47th week (Sep. 26）and the 52th week (Nov. 1) atter anthesis.
