# Supplementary material for: Integrative Metabolic and Transcriptomic Profiling in Camellia oleifera and Camellia meiocarpa Uncover Potential Mechanisms That Govern Triacylglycerol Degradation during Seed Desiccation
Source: Plants (Basel). 2023 Jul 8;12(14):2591. doi: 10.3390/plants12142591 (PMC10385360; doi:10.3390/plants12142591)
Supplement: Supplementary file 1 [file plants-12-02591-s001.zip › Supplementary Table S1.pdf]

Supplementary Table S1. Fatty acid content changes during seed desiccation in *C. meiocarpa var Qingguo, C. meiocarpa var Hongguo*, and *C. oleifera cv Min 43*.

Germplasm	Weeks after anthesis	16:0	16:1$^{\Delta 11}$	18:0	18:1$^{\Delta 9}$	18:1$^{\Delta 11}$	18:2$^{\Delta 9,12}$	18:2$^{\Delta 9,12,15}$	20:0	20:1$^{\Delta 13}$
QG	46	2.30 ± 0.30	0.03 ± 0.02	0.21 ± 0.03	10.89 ± 1.14	0.49 ± 0.06	3.52 ± 0.44	0.12 ± 0.01	0.35 ± 0.03	0.03 ± 0.03
	47	1.92 ± 0.07	0.05 ± 0.00	0.30 ± 0.08	11.83 ± 1.39	0.58 ± 0.07	3.40 ± 0.36	0.12 ± 0.03	0.17 ± 0.04	0.03 ± 0.02
	48	2.34 ± 0.23	0.04 ± 0.01	0.30 ± 0.03	14.96 ± 1.25	0.58 ± 0.06	3.55 ± 0.39	0.14 ± 0.01	0.23 ± 0.02	0.05 ± 0.01
	49	3.44 ± 0.36	0.06 ± 0.00	0.48 ± 0.15	20.69 ± 1.32	0.85 ± 0.07	5.11 ± 0.38	0.23 ± 0.01	0.26 ± 0.02	0.03 ± 0.03
	50	3.17 ± 0.50	0.01 ± 0.02	0.72 ± 0.11	24.90 ± 3.91	0.55 ± 0.07	3.98 ± 0.61	0.16 ± 0.02	0.16 ± 0.02	0.07 ± 0.01
	51	4.01 ± 0.65	0.04 ± 0.01	0.47 ± 0.06	34.25 ± 4.65	0.56 ± 0.36	4.42 ± 0.55	0.26 ± 0.03	0.19 ± 0.02	0.09 ± 0.01
	52	4.09 ± 0.20	0.04 ± 0.00	1.40 ± 0.08	40.08 ± 1.98	0.06 ± 0.05	3.71 ± 0.17	0.22 ± 0.02	0.15 ± 0.02	0.10 ± 0.00
	53	5.00 ± 0.73	0.06 ± 0.01	1.31 ± 0.36	46.64 ± 9.66	0.36 ± 0.52	4.29 ± 0.43	0.27 ± 0.09	0.19 ± 0.05	0.09 ± 0.03
	54	3.82 ± 0.53	0.04 ± 0.00	1.09 ± 0.12	34.32 ± 3.80	0.08 ± 0.07	3.78 ± 0.81	0.22 ± 0.02	0.16 ± 0.02	0.06 ± 0.02
HG	46	2.05 ±0.46	0.01 ± 0.02	0.30 ± 0.13	11.56 ± 1.90	0.51 ± 0.08	3.55 ± 0.65	0.12 ± 0.02	0.30 ± 0.05	0.03 ± 0.03
	47	4.17± 0.38	0.06 ± 0.01	0.48 ± 0.04	24.99 ± 2.26	0.81 ± 0.08	4.35 ± 0.43	0.19 ± 0.02	0.23 ± 0.02	0.07 ± 0.00
	48	3.12 ± 0.42	0.05 ± 0.01	0.59 ± 0.07	26.19 ± 3.18	0.67 ± 0.08	3.10 ± 0.39	0.18 ± 0.02	0.00 ± 0.00	0.06 ± 0.01
	49	3.82 ± 0.66	0.06 ± 0.01	0.53 ± 0.09	26.32 ± 4.31	0.78 ± 0.12	4.25 ± 0.79	0.18 ± 0.03	0.21 ± 0.03	0.06 ± 0.00
	50	3.94 ± 1.75	0.06 ± 0.03	0.43 ± 0.19	22.40 ± 9.93	0.92 ± 0.42	6.71 ± 2.90	0.24 ± 0.11	0.32 ± 0.15	0.04 ± 0.04
	51	3.40 ± 0.22	0.05 ± 0.01	0.59 ± 0.04	23.58 ± 1.25	0.71 ± 0. 06	5.78 ± 0.51	0.18 ± 0.01	0.25 ± 0.02	0.05 ± 0.01
	52	3.86 ± 0.33	0.00 ± 0.00	0.94 ± 0.07	29.23 ± 2.27	0.51 ± 0.32	3.94 ± 0.35	0.19 ± 0.02	0.18 ± 0.03	0.07 ± 0.01
	53	3.50 ± 0.30	0.00 ± 0.00	1.18 ± 0.11	32.77 ± 3.69	0.50 ± 0.32	4.21 ± 0.33	0.24 ± 0.02	0.19 ± 0.01	0.06 ± 0.01
	54	3.20 ± 0.76	0.04 ± 0.00	1.23 ± 0.16	35.06 ± 4.93	0.07 ± 0.06	3.53 ± 0.49	0.23 ± 0.03	0.23 ± 0.03	0.07 ± 0.01
M43	46	3.54 ± 0.02	0.06 ± 0.00	0.40 ± 0.01	23.81 ± 0.14	0.89 ± 0.01	5.25 ± 0.07	0.17 ± 0.00	0.24 ± 0.01	0.07 ± 0.00
	47	3.83 ± 0.26	0.04 ± 0.00	0.34 ± 0.03	19.68 ± 1.34	0.70 ± 0.04	3.86 ± 0.28	0.19 ± 0.01	0.39 ± 0.04	0.06 ± 0.01
	48	4.02 ± 0.62	0.06 ± 0.00	0.31 ± 0.10	24.83 ± 7.36	0.91 ± 0.09	5.11 ± 0.53	0.20 ± 0.05	0.32 ± 0.01	0.07 ± 0.01
	49	4.08 ± 0.25	0.05 ± 0.00	0.38 ± 0.02	28.79 ± 1.78	0.87 ± 0.05	4.85 ± 0.32	0.23 ± 0.01	0.27 ± 0.01	0.07 ± 0.00

	50	3.27 ± 0.21	0.01 ± 0.02	0.38 ± 0.03	25.43 ± 1.54	0.64 ± 0.02	3.66 ± 0.18	0.22 ± 0.01	0.23 ± 0.01	0.07 ± 0.02
	51	4.58 ± 1.78	0.03 ± 0.03	0.69 ± 0.43	35.79 ± 10.94	0.89 ± 0.28	5.21 ± 1.60	0.27 ± 0.07	0.22 ± 0.05	0.06 ± 0.01
	52	5.51 ± 2.28	0.00 ± 0.00	0.83 ± 0.29	48.00 ± 20.98	0.34 ± 0.27	6.01 ± 2.67	0.39 ± 0.17	0.29 ± 0.13	0.07 ± 0.07
	53	3.96 ± 1.64	0.04 ± 0.03	0.66 ± 0.31	37.92 ± 18.22	0.18 ± 0.21	2.95 ± 1.41	0.19 ± 0.09	0.13 ± 0.07	0.08 ± 0.04
	54	3.59 ± 0.59	0.00 ± 0.00	0.67 ± 0.21	35.18 ± 10.53	0.02 ± 0.03	3.14 ± 0.86	0.25 ± 0.07	0.13 ± 0.03	0.06 ± 0.01

QG, *C. meiocarpa var Qingguo*; HG, *C. meiocarpa var Hongguo*; M43, *C. oleifera cv Min 43*. Data were expressed as mean ± SD (n = 3).
