# Supplementary material for: Integrative Metabolic and Transcriptomic Profiling in Camellia oleifera and Camellia meiocarpa Uncover Potential Mechanisms That Govern Triacylglycerol Degradation during Seed Desiccation
Source: Plants (Basel). 2023 Jul 8;12(14):2591. doi: 10.3390/plants12142591 (PMC10385360; doi:10.3390/plants12142591)
Supplement: Supplementary file 1 [file plants-12-02591-s001.zip › Supplementary Table S11.pdf]

Supplementary Table S11. Selected genes and their primer sequences for qRT-PCR analysis

	Gene ID in HG	Gene ID in Min 43	Forward primer	Reverse primer
Control	GAPDH TRINITY_DN25806_c1_g3_i1_1	GAPDH TRINITY_DN35918_c1_g1_i1_2	5’-GGTTTGGCATTGTTGAGGGT-3’	5’-CCTCTCCAGTCCTTGCTTGA-3’
1	TRINITY_DN20394_c0_g1_i1_2	TRINITY_DN24587_c0_g1_i1_1	5’-GGCAAGACTTGTCCGAGGGA-3’	ACTCGCAGCATCGGTTGTCA-3’
2	TRINITY_DN25522_c0_g1_i1_1	TRINITY_DN32370_c0_g1_i4_2	5’-TTCAAGCCTCCTCGATGCCA-3’	5’-TTGGTGGGTCTCATGGCAGA-3’
3	TRINITY_DN21501_c0_g1_i1_1	TRINITY_DN26190_c1_g1_i1_1	5’-ACCCGATGCACCCGAAATCA-3’	5’-CCGTTGAGCCTCGTTCTCCA-3’
4	TRINITY_DN23188_c1_g1_i1_1	TRINITY_DN24343_c0_g2_i3_1	5’-AGGATGCCACGTAGCTCTGG-3’	5’-TGAAGGCGTCGAAGGTGAGG-3’
5	TRINITY_DN29729_c0_g2_i1_1	TRINITY_DN23466_c2_g1_i2_2	5’-ACTTCTGCCATGATCGTCCCA-3’	5’-CCTCGATCTGACATCTGCACG-3’
6	TRINITY_DN28894_c0_g2_i1_1	TRINITY_DN35399_c0_g3_i1_2	5’-TCATGAGACTCAAGCAAACGCA-3’	5’-AGTGCTGGAGGGATTGTCTGT-3’
7	TRINITY_DN17413_c0_g1_i1_2	TRINITY_DN30341_c0_g1_i3_1	5’-GCATCCCCCATCAGTAAG-3’	5’-TTGCTCCCCAGATTCC-3’
8	TRINITY_DN4285_c0_g1_i1_2	TRINITY_DN21633_c0_g1_i1_1	5’-CCAGCACATGAGGAGGCGAT-3’	5’-GCCTCTCCTCTCTCCTCATCGT-3’
